# Preoperative CT-based radiomics nomogram for progression-free survival prediction in pediatric posterior mediastinal malignancies

**DOI:** 10.3389/fonc.2025.1586980

**Published:** 2025-04-11

**Authors:** Shucheng Bi, Chenghao Chen, Jie Yu, Ting Yang, Jihang Sun, Zunying Hu, Qi Zeng, Yun Peng

**Affiliations:** ^1^ Department of Radiology, Ministry of Education (MOE) Key Laboratory of Major Diseases in Children, Beijing Children’s Hospital, Capital Medical University, National Center for Children’s Health, Beijing, China; ^2^ Department of Thoracic Surgery, Beijing Children’s Hospital, Capital Medical University, National Center for Children’s Health, Beijing, China

**Keywords:** pediatric, mediastinal, malignant, radiomics, progression-free survival, CT

## Abstract

**Background:**

Progression-free survival (PFS) prediction plays a pivotal role in developing personalized treatment strategies and ensuring favorable long-term outcomes in pediatric posterior mediastinal malignant tumors. This study developed and validated the first preoperative contrast-enhanced computed tomography (CT)-based radiomics nomogram to forecast PFS in posterior mediastinal malignancies patients. The aim was to provide a clinically applicable prognostic tool to stratify high-risk populations.

**Methods:**

Medical data from 306 patients with posterior mediastinal malignancies were analyzed retrospectively and randomly divided into training (n = 215) and test sets (n = 91). The clinical model was built using conventional clinical data and CT signs. Selection of the radiomic features was performed using maximum relevance minimum redundancy and the least absolute shrinkage and selection operator. To overcome class imbalance, the synthetic minority over-sampling technique was used in the training set. Radiomics signature was derived using logistic regression algorithm, and we developed a nomogram by integrating the clinical model and the radiomics signature. The predictive efficiency of the nomogram was assessed using the area under the curve (AUC), brier score (BS), decision curve analysis, and calibration.

**Results:**

The Ki-67 index and metastasis were identified as independent predictors, with the test set achieving an AUC of 0.82 (0.647–0.964) and a BS of 0.21 (0.181–0.239). Nineteen radiomics features most relevant to PFS were retained, with the logistic regression algorithm achieving an AUC of 0.77 (0.589–0.896) and a BS of 0.26 (0.215–0.292) in the test set. The radiomics nomogram demonstrated best predictive capability in the test set, achieving an AUC of 0.87 (0.733–0.968) and a BS of 0.22 (0.177–0.255), compared with remaining prediction models. Both calibration curves and decision curve analysis demonstrated good fit and clinical benefit.

**Conclusions:**

Our contrast-enhanced CT-based radiomics nomogram may be a dependable, precise, and noninvasive prognostic tool to predict PFS in pediatric posterior mediastinal malignancies preoperatively.

## 1 Introduction

Posterior mediastinal malignant tumors (PMMTs) constitute roughly 1/3 of all pediatric mediastinal masses (1, 2). PMMTs include various subtypes (3), with approximately 90% being neurogenic, and neuroblastoma (NB) is the most prevalent tumor (4, 5). At the time of first diagnosis, approximately 40% of children with PMMTs, due to their high heterogeneity, have locally advanced disease, or bone, bone marrow, or other metastases, which lead to a generally poor prognosis (6–8). Notably, children with intermediate- and high-risk NB have an unsatisfactory 5-year survival rate (9, 10). Accurate prediction of progression-free survival (PFS) for PMMTs could impact the choice to pursue intensive management. Currently, studies on NB suggest that the international NB staging system, namely involving age and pathological grading, can effectively predict 2-year PFS (11, 12). However, owing to the high heterogeneity of PMMTs, there is still a lack of unified standards and reliable prognostic biomarkers. Therefore, there is an urgent clinical need for an effective prognostic tool that integrates multiple factors influencing PFS.

Radiomics, an emerging field in medical imaging, presents a promising alternative to traditional visual inspection by radiologists (13–15). By analyzing extensive quantitative data from various types of medical imaging, radiomics accurately characterizes the heterogeneity and biological behavior of tumors (16, 17). Lately, radiomics has been widely applied to help with tumor diagnosis, distinguishing between different tumors, staging, tracking disease progression, and assessing treatment outcomes (18, 19). Radiomics has been used to forecast the overall survival of pediatric diffuse midline gliomas (20), and the PFS of NB patients (21) and the event-free survival of patients with hepatoblastoma (22). Furthermore, CT-based deep learning models have been very effective in identifying advanced-stage pulmonary tuberculosis in children (23) and differentiating pediatric non-Wilms tumors (24).

From the authors’ perspective, the implementation of radiomics in predicting PFS in patients with PMMTs remains limited and is not yet widely applied. Thus, our objective was to construct a radiomics nomogram using contrast-enhanced CT to predict PFS in pediatric patients with PMMTs preoperatively.

## 2 Materials and methods

### 2.1 Patients

**Figure 1:**
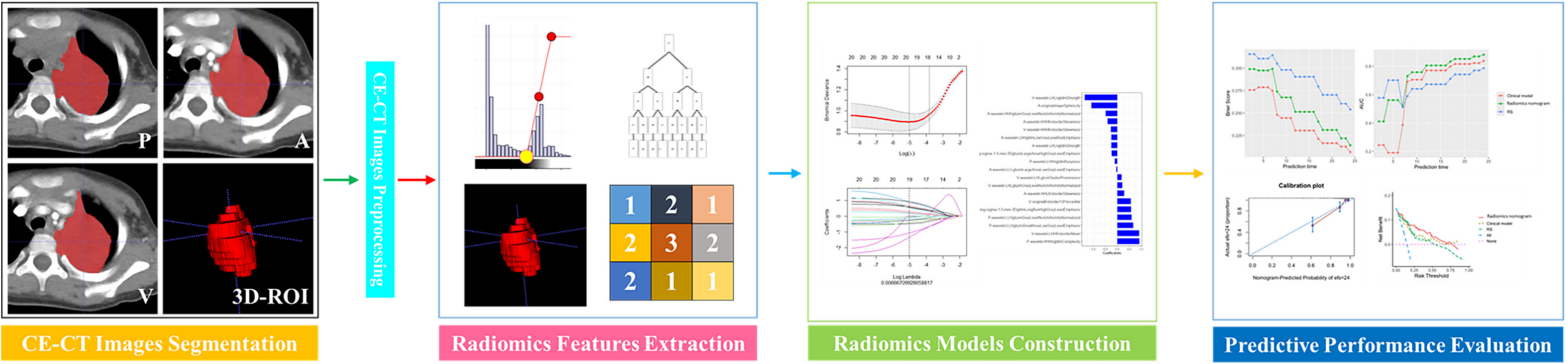
The radiomics approach used in this study.

Approval for this retrospective study was granted by our institutional ethical committee, and informed consent was not required. This study involved data for pediatric PMMT patients who were treated between February 2013 and December 2022. The inclusion criteria were as follows: 1) malignancies in the posterior mediastinum confirmed pathologically after surgery, 2) routine contrast-enhanced CT within 15 days prior to treatment, and 3) PMMTs in patients who were ≤ 18 years of age at diagnosis. Patients with PMMTs were excluded if their clinical or imaging data were incomplete. Ultimately, data for 306 patients with PMMTs were incorporated into this retrospective analysis. In a 7:3 proportion, all PMMTs were assigned to training (n = 215) and test sets (n = 91) randomly, in accordance with the Transparent Reporting of a multivariable prediction model for Individual Prognosis Or Diagnosis (TRIPOD) statement (25). The follow-up plan for postoperative pediatric patients was as follows: follow-up visits are scheduled at 1, 3, 6, 12, and 24 months post-surgery, primarily involving imaging examinations to monitor for tumor progression. The follow-up period ended on December 31, 2024. The procedure for radiomics in this study is depicted in **Figure 1**.

### 2.2 Collection of contrast-enhanced CT images

Preoperative standard CT images of PMMTs comprised precontrast, arterial, and venous phase images, which were collected using GE Discovery CT750 HD and GE Healthcare 64/16 slice spiral CT units (General Electric Company, Cincinnati, OH, USA). Imaging included the chest, with a 5-mm slice thickness and no interslice gap. The procedure involved instructing the patient to lie supine, followed by plain CT to determine the location of the lesion, and then contrast-enhanced CT. Contrast imaging included both arterial and venous phases. The contrast medium was iodixanol at 300 mgI/ml, administered in doses ranging from 1.1 to 1.6 ml/kg. Arterial phase images were acquired 15–18 seconds after the contrast injection, and venous phase images were obtained 45–55 seconds after injection.

### 2.3 Review of conventional CT Signs

**Figure 2:**
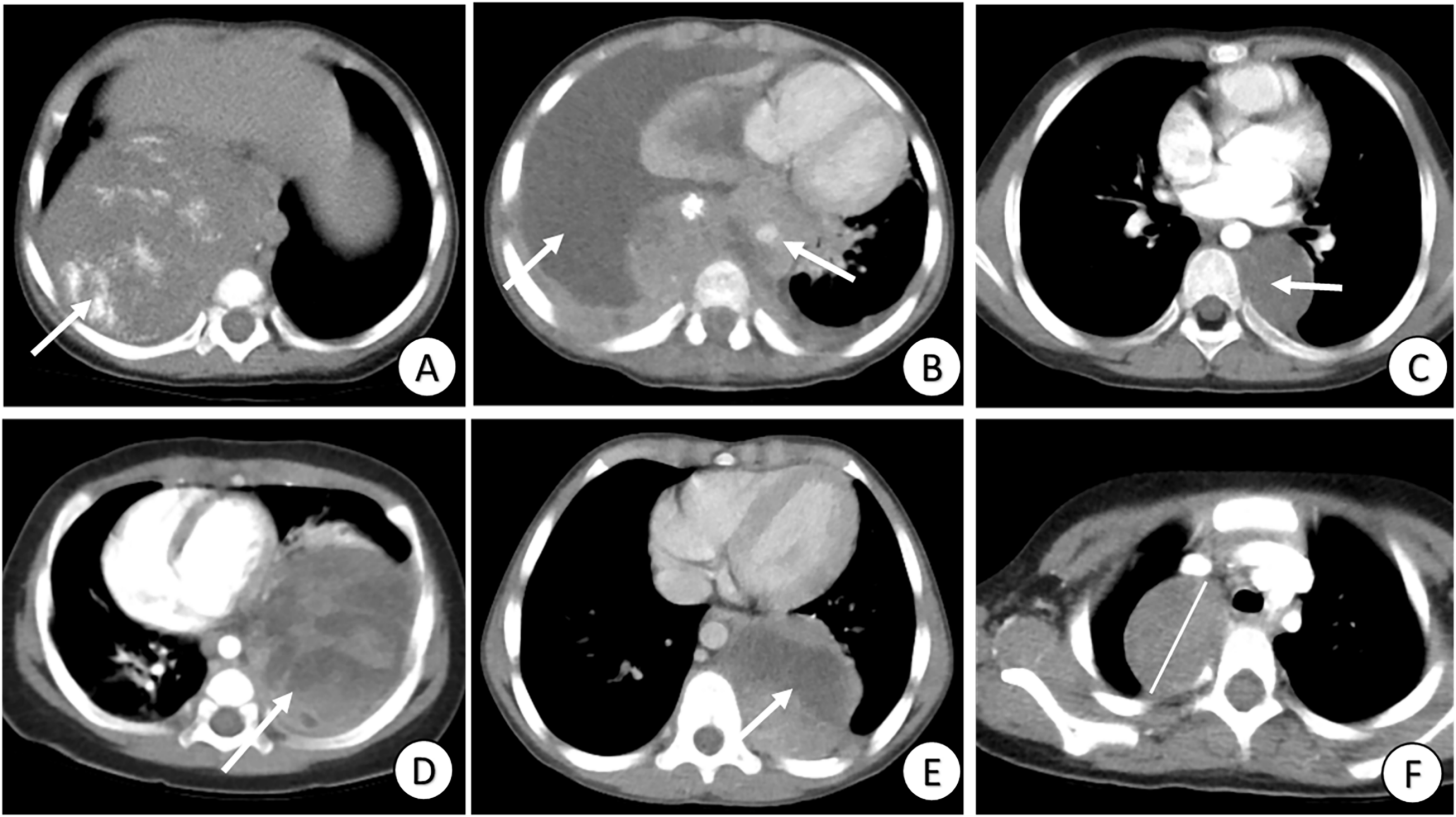
Review of conventional clinical data and CT signs. **(A)** Tumor with calcification (arrow); **(B)** Tumor with pleural effusion, infiltration across the midline and vascular wrapping (arrows); **(C)** Tumor with well-defined margins and heterogeneity (arrow); **(D)** Tumor with heterogeneity and nonuniform enhancement (arrow); **(E)** Tumor with necrosis (arrow); and **(F)** Tumor with maximal diameter = 4.3 cm (line).

Three radiologists, with 7, 10, and 12 years’ experience, independently evaluated all-phase CT images of the PMMTs, respectively. The radiologists had no access to the patients’ clinical data or pathological details. In instances of disagreement regarding conventional CT findings, the final decisions were made through discussion and consensus among the radiologists. The CT features were categorized as follows (**Figure 2**): 1) maximal diameter, 2) location, 3) heterogeneity, 4) margin (well-defined or ill-defined), 5) infiltration across the midline, 6) vascular wrapping, 7) pleural effusion, 8) calcification, 9) necrosis, 10) enhancement (uniform or nonuniform), and 11) metastasis. Features 2–11 were categorized retrospectively as either negative or positive. Researchers retrospectively collected clinical data, namely gender, age, Ki-67 index, pathological type, and information on complete resection.

### 2.4 Image segmentation and radiomic feature extraction

Using the open-source ITK-SNAP software (version 4.2.0) (26), radiologist A manually segmented the neoplastic three-dimensional region of interest (3D-ROI). The area included the entire tumor parenchyma and excluded surrounding inflammation, necrosis, pleural effusion, atelectatic lung tissue, blood vessels, and bone. Arterial and venous phase images were used to aid in accurate segmentation when the boundary was not clearly visible on precontrast images.

To measure intraobserver agreement, radiologist A manually segmented 40 randomly selected cases after a period of 2 weeks. Radiologist B independently outlined the 3D-ROIs for an equivalent number of patients to assess interobserver agreement. The clinical and histopathological details were not disclosed to either radiologist. By calculating intra-/interobserver correlation coefficients, the consistency of each radiomics feature was assessed regarding intra-/interobserver agreement.

Preprocessing steps were implemented to minimize feature variability and address inconsistent intensity because of varying scanning sequences and parameters. To strengthen the signal-to-noise ratio in texture analysis, gray-level quantization was used to decrease the gray levels. To achieve uniform voxel spacing, cubic interpolation was applied, adjusting the 3D-ROIs to an isotropic resolution (voxel size = 1 × 1 × 1 mm³).

3D Slicer (version 5.8.0) (27), an open-source free software, offers comprehensive and flexible tools for multimodal image processing and radiomics feature extraction, positioning it as an essential platform for medical image analysis research. Radiomic features, namely shape, first-order, and texture features (such as neighboring gray tone difference matrix, gray level run length matrix, gray level co-occurrence matrix, gray level dependence matrix, gray level size zone matrix) were extracted from 3D-ROIs for all-phase CT images. A wavelet transform was subsequently applied to emphasize both high- and low-frequency information for further analysis. Feature calculations were performed in accordance with the guidelines set forth by the Image Biomarker Standardisation Initiative (28).

### 2.5 Construction and evaluation of different models

**Figure 3:**
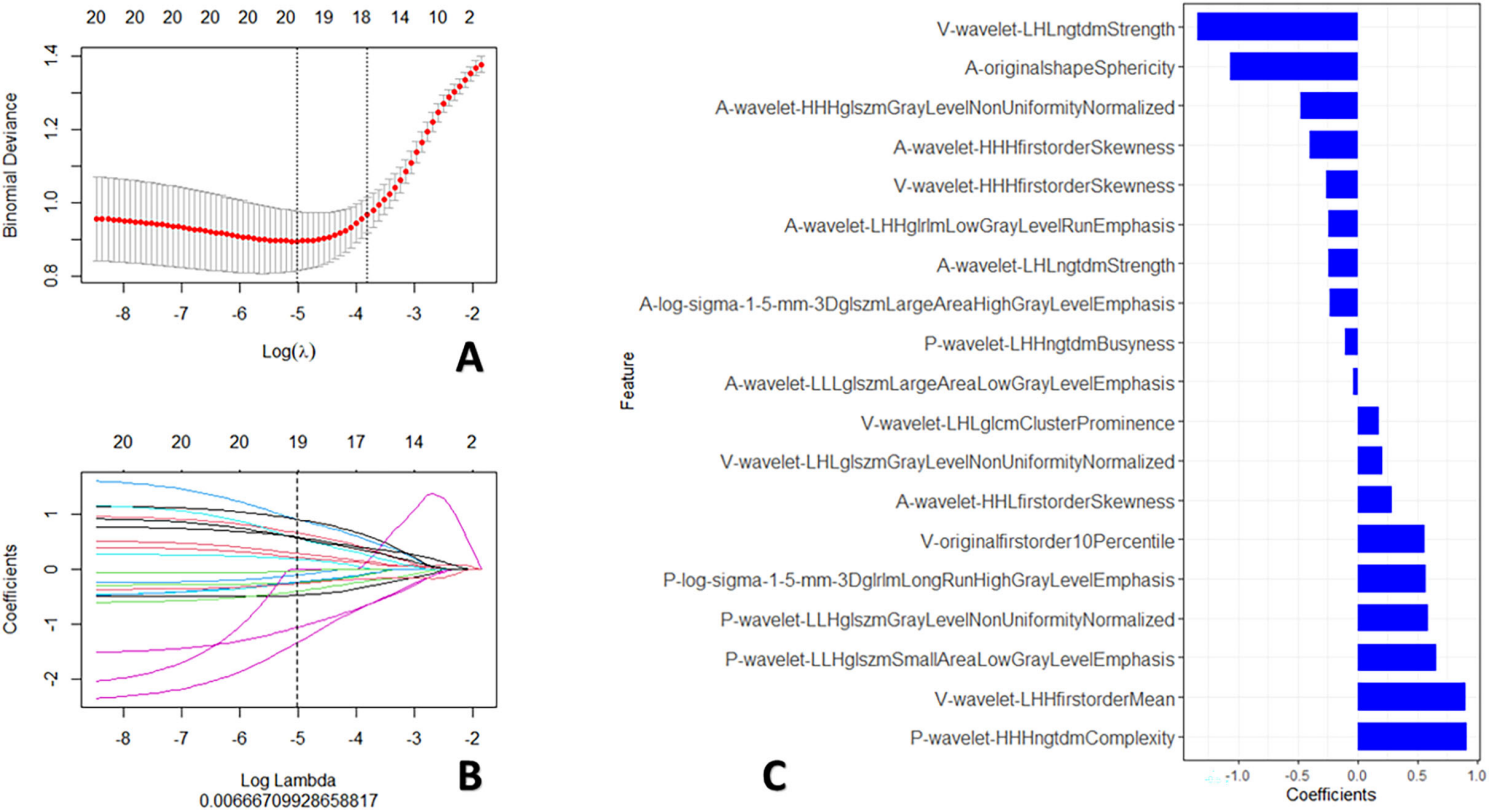
Procedures for radiomics feature screening. **(A)** Procedure for selecting the fine-tuning parameter (λ); **(B)** Graphed coefficients against ln (λ); and **(C)** The 19 chosen radiomics features.

The standardization of all radiomic features from the all-phase images was performed using the z-score method. To address imbalanced data characteristics among the groups (29), the synthetic minority over-sampling technique (SMOTE) was used to process all radiomic features in the training set for subsequent analysis. Then, three procedures were used to identify the most predictive features to predict the survival of children with PMMTs. First, using the maximum relevance minimum redundancy approach, the 20 most relevant radiomic features were retained, while irrelevant and redundant features were removed. Next, using the least absolute shrinkage and selection operator (LASSO), the most predictive features were identified. Integrating the chosen radiomics features with their LASSO coefficients linearly, a Rad-score was built for each case. Finally, R software (www.r-project.org) was utilized to construct the radiomics signature (RS) using logistic regression algorithm, as illustrated in **Figure 3**.

The conventional clinical data and CT signs were examined by the Chi-square test and Wilcoxon’s test. In the univariate analysis, factors with p-values < 0.05 were identified and chosen for use in the multivariate analysis to construct the clinical model. Ultimately, to create a dependable radiomics nomogram, multivariate analysis was used to integrate the RS and clinical model.

The ability of all models to predict PFS in patients with PMMTs was assessed in the training and test sets using the area under curve (AUC), brier score (BS), and calibration curves. Decision curve analysis was used in the training and test sets to assess the nomogram’s applicability in clinical practice.

### 2.6 Statistical analysis

A t-test was used for continuous variables, while the chi-square or Fisher’s exact test was used for categorical variables. Subsequently, the conventional clinical data and CT signs were evaluated by univariate and multivariate analyses. Intra-/interobserver correlation coefficients ≥ 0.80 indicated strong agreement. The accuracy of all models was evaluated using the BS. Differences in the calibration curves were compared using the Hosmer–Lemeshow test. Statistical analyses were performed using R statistical software (version 4.4.2), with statistical significance defined as p < 0.05.

## 3 Results

### 3.1 Conventional clinical data and CT signs

The medical baseline data for all PMMT patients, including conventional clinical data and CT signs, are shown in **Table 1**. Necrotic lesions constituted > 10% of all neoplasms. In the training set, 32 of 215 (14.9%) patients experienced progression, with a median follow-up time of 7.23 months (interquartile range: 4.45–18.08). In the test set, 13 of 91 (14.3%) patients experienced progression, with a median follow-up time of 8.57 months (interquartile range: 7.17–16.33).

**Table 1:** Conventional clinical data and CT signs in pediatric patients with PMMTs.

		Training set (n=215)	Test set (n=91)	P
Progression	No	183	78	1.000
	Yes	32	13	
Progression-Free Survival* (months)		7.23 (4.45,18.08)	8.57 (7.17,16.33	<0.001
Gender	Male	88	54	0.003
	Female	127	37	
Age* (months)		39.00 (16.00,65.00)	41.00 (21.00,72.00)	0.429
Ki-67 index*		8.00 (3.00, 40.00)	5.00 (3.00,20.00)	<0.001
Pathological type	NB	86	28	0.114
	non-NB	129	63	
Complete resection	+	175	84	0.108
	–	40	7	
Maximal Diameter* (cm)		6.40 (4.20,8.90)	6.10 (4.50,8.40)	0.704
Location	L	116	50	0.874
	R	99	41	
Heterogeneity	–	88	41	0.504
	+	127	50	
Margin	Well-defined	68	30	0.818
	III-defined	147	61	
Infiltration Across the Midline	–	126	13	0.013
	+	89	67	
Vascular Wrapping	–	98	52	0.064
	+	117	39	
Pleural Effusion	–	128	64	0.074
	+	87	27	
Calcification	–	75	40	0.134
	+	140	52	
Necrosis	–	153	74	0.064
	+	62	17	
Enhancement	Uniform	88	41	0.504
	Non-uniform	127	50	
Metastasis	–	165	74	0.376
	+	50	17	

① Continuous variables* are presented as median (Q1, Q3) and compared using an independent t-test.

② Categorical variables were compared using Fisher's exact test or chi-square test.

The conventional clinical data and CT signs were incorporated into the next step of the study (**Table 2**). Univariate analysis indicated that the risk factors for poor survival were Ki-67 index, maximal diameter, infiltration across the midline, vascular wrapping, calcification, necrosis, and metastasis (all, p < 0.05). On the basis of the multivariate analysis, Ki-67 index and metastasis (both, p < 0.05) were used to develop the clinical model, which achieved an AUC of 0.82 (0.647–0.964) and a BS of 0.21 (0.181–0.239) in the test set.

**Table 2:** Results of the univariate and multivariate analyses of conventional clinical data and CT signs.

Variables	Univariate Analysis		P	Multivariable Analysis		P
	OR	95%CI		OR	95%CI	
Gender	0.649	(0.305-1.378)	0.260			
Age	1.008	(0.999-1.017)	0.077			
Ki-67 index	1.037	(1.023-1.052)	<0.001	1.037	(1.021-1.053)	<0.001
Pathological type	0.718	(0.338-1.529)	0.391			
Complete resection	2.318	(0.997-5.390)	0.051			
Maximal Diameter	1.122	(1.005-1.253)	0.040	0.958	(0.800-1.147)	0.640
Location	1.040	(0.490-2.208)	0.919			
Heterogeneity	1.941	(0.851-4.426)	0.115			
Margin	2.220	(0.868-5.678)	0.096			
Infiltration Across the Midline	3.229	(1.467-7.105)	0.004	0.454	(0.113-1.826)	0.266
Vascular Wrapping	2.420	(1.062-5.512)	0.035	0.972	(0.206-4.595)	0.971
Pleural Effusion	1.830	(0.859-3.895)	0.117			
Calcification	3.345	(1.231-9.090)	0.018	0.374	(0.115-1.215)	0.102
Necrosis	2.553	(1.183-5.511)	0.017	1.159	(0.359-3.739)	0.805
Enhancement	1.941	(0.851-4.426)	0.115			
Metastasis	6.067	(2.738-13.446)	<0.001	0.200	(0.073-0.546)	0.002
Rad_score	1.986	(1.530-2.579)	<0.001	1.189	(1.363-2.428)	<0.001

OR, odd ratio; CI, confidence interval.

### 3.2 Establishment and performance of prognostic models

**Figure 4:**
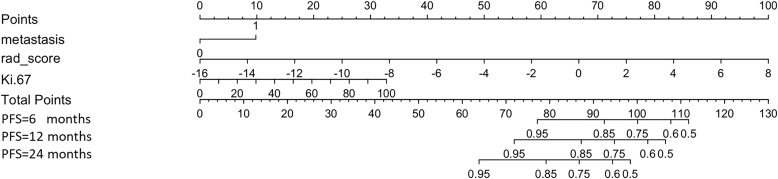
Radiomics nomogram. The nomogram combines metastasis, rad-score, and Ki-67 to calculate a total score, which is used to predict the patient’s progression-free survival (PFS) at 6, 12, and 24 months.

**Figure 5:**
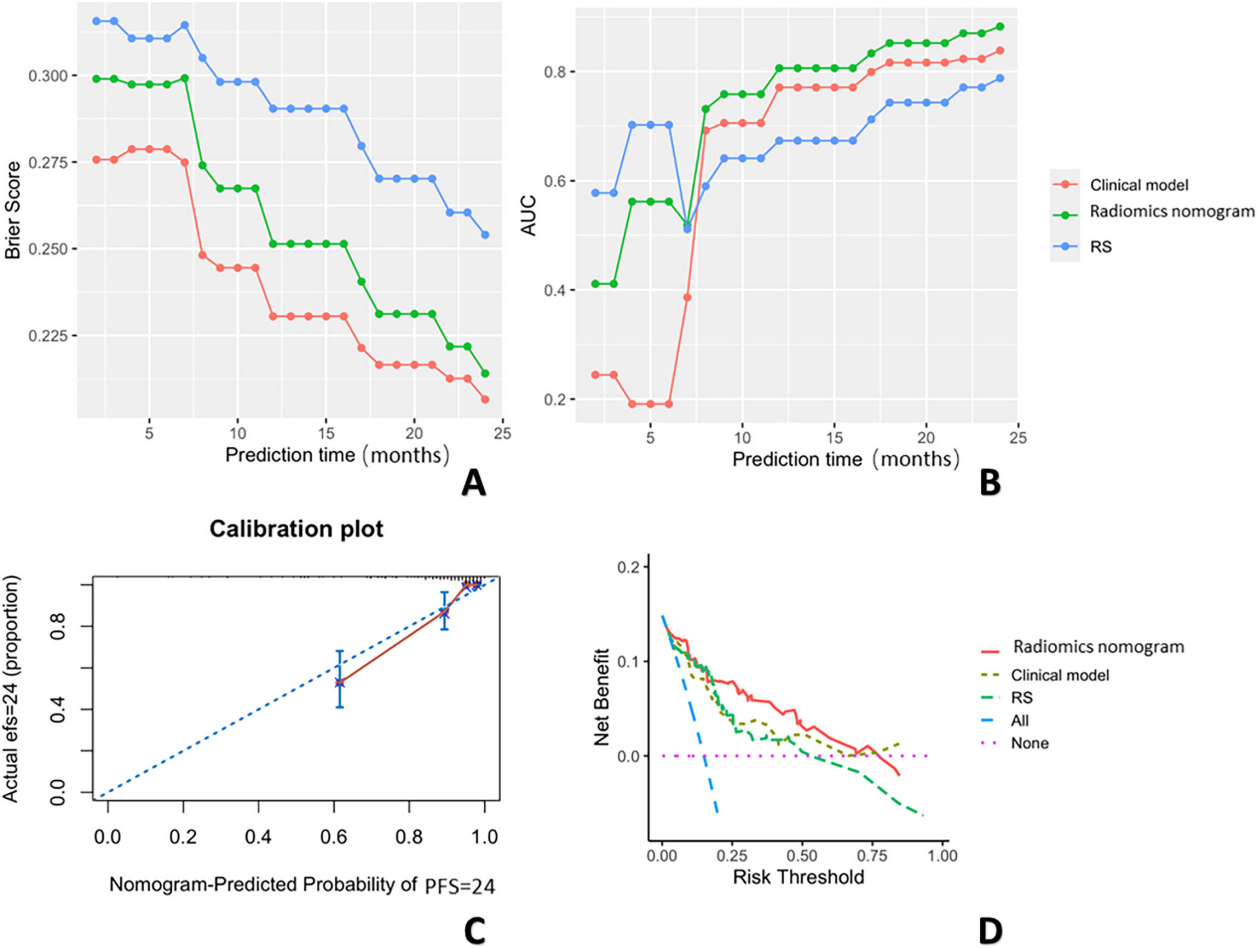
The BSs **(A)** across three prediction models over 24 months. This figure shows the changes in BS values over a 24-month period for three prediction models: the clinical model (red), the radiomics nomogram (green), and the RS (blue). The x-axis represents the observation time points (in months), and the y-axis represents the BS values. The BS trends differ across the models. The AUCs **(B)** are shown across the three prediction models over 24 months. This figure shows the changes in AUC values over a 24-month period for three prediction models: the clinical model (red), radiomics nomogram (green), and the RS (blue). The x-axis represents the prediction time points (in months), and the y-axis represents the AUC values. The calibration curves **(C)**, and decision curve analysis results **(D)** are also shown.

Using the maximum relevance minimum redundancy and LASSO algorithms, 19 radiomics features were preserved, as shown in **Figure 3**. The RS was built using logistic regression, with an AUC of 0.77 (0.589–0.896) and a BS of 0.26 (**Table 3**). Then, the radiomics nomogram was constructed by integrating the RS with the clinical model (**Figure 4**). The predictive performance of the radiomics nomogram is showed in **Table 3**. The radiomics nomogram demonstrated outstanding PFS prediction performance, with an AUC of 0.87 (0.733–0.968) and a BS of 0.22 (0.177–0.255) (**Figures 5A, B**) in the test set. **Figures 5C, D** shows that the calibration curves and decision curve analysis results indicated good fit and clinical benefit.

**Table 3:** Predictive performance of various models to assess progression-free survival.

	Brier score		95%CI	AUC	95%CI
Clinical Model	Training	0.27	(0.237-0.286)	0.85	(0.789-0.909)
	Test	0.21	(0.181-0.239)	0.82	(0.647-0.964)
Radiomics Signature	Training	0.24	(0.208-0.259)	0.87	(0.814-0.912)
	Test	0.26	(0.215-0.292)	0.77	(0.589-0.896)
Radiomics Nomogram	Training	0.24	(0.212-0.266)	0.91	(0.858-0.943)
	Test	0.22	(0.177-0.255)	0.87	(0.733-0.968)

## 4 Discussion

In this study, we developed a radiomics nomogram using contrast-enhanced CT to provide a dependable, precise, and noninvasive prognostic tool to predict PFS in patients with PMMTs. To our knowledge, this study is one of the first to use contrast-enhanced CT-based radiomics to assess the PFS of pediatric patients with PMMTs. By integrating both clinical data and RS, we constructed a radiomics nomogram that achieved promising results, with an AUC of 0.87 (0.733–0.968) in the test set. These findings demonstrate that our contrast-enhanced CT-based radiomics nomogram is an effective prognostic tool to preoperatively predict PFS in pediatric patients with PMMTs. As a result, the nomogram may help clinicians personalize treatment strategies and improve the long-term prognosis of pediatric patients with PMMTs.

Currently, the PFS prediction of patients with malignant tumors in clinical practice is primarily grounded in traditional clinical metrics and conventional CT findings. In our study, we retrospectively analyzed data from 306 pediatric patients with PMMTs. The clinical model, which included the Ki-67 index and metastasis as significant predictors identified through univariate and multivariate analyses (all, p < 0.05), achieved an AUC of 0.82 (0.647–0.964) in the test set. Ki-67 is an important cell proliferation marker that reflects tumor proliferative activity and is commonly used to assess the growth rate and malignancy of tumors (30). The presence of metastasis is a key factor in tumor prognosis (31), directly affecting a patient’s survival. The clinical model showed strong performance in predicting PFS and effectively distinguishing between patients with varying survival durations. However, the clinical model included only Ki-67 and metastasis, potentially overlooking other relevant tumor features and molecular characteristics. Therefore, incorporating additional types of data is essential to further optimize the model and improve the accuracy of PFS prediction for malignant tumors.

Previous studies have highlighted the capability of radiomics to assess the biological behavior and prognosis of various malignancies. For example, Sui et al. created a radiomics model with an AUC of 0.89 using positron emission tomography/CT to predict prognosis in patients with hepatocellular carcinoma (32). To develop a prognostic model for locally advanced gastric cancer patients, Li et al. designed a CT-based radiomics model with an AUC of 0.73 (33). These studies emphasize the substantial potential of radiomics to predict survival across malignancies. In this research, we constructed an RS from contrast-enhanced CT images, enabling precise presurgical prediction of PFS and achieving an AUC of 0.77 (0.589–0.896) in the test set. Although the feasibility of radiomics has been demonstrated, this method has certain limitations compared with clinical models, particularly for stability and reliability. Therefore, relying solely on radiomics may not fully enhance predictive accuracy and overlooks the significant contribution of clinical factors in PFS prediction.

To overcome the limitations of relying solely on radiomics, this study further explored a nomogram that integrated both a clinical model and radiomics. By combining radiomics with the clinical model (Ki-67 and metastatic status), the nomogram provides a comprehensive fusion of tumor imaging characteristics and biological behavior, leveraging the complementary strengths of both data types. Previous studies have shown the efficacy of nomograms to predict survival outcomes by incorporating clinical and radiomics features. Liu et al. developed a nomogram (C-index = 0.78) that integrated radiomics and clinical data to predict overall survival in patients with hepatocellular carcinoma after hepatectomy, showing improved predictive performance (34). In the present study, the nomogram achieved an AUC of 0.87 (0.733–0.968) in the test set, which was a significantly higher AUC compared with the radiomics approach, and a BS of 0.22, showing notable improvement over radiomics. These results demonstrate that the nomogram substantially enhances the accuracy and stability of PFS prediction in malignancies. By addressing the limitations of single-source data, the nomogram offers a comprehensive and reliable prognostic assessment for clinical decision-making.

Several limitations of this study must be acknowledged. First, even with strict exclusion and inclusion criteria, selection bias cannot be entirely ruled out because of the retrospective study design. Second, segmentation of all PMMTs was used to outline the ROI manually, potentially introducing variability. Future studies should consider a semiautomatic segmentation method for more precise ROI delineation. Third, although we included data for 306 patients, this was a relatively limited sample size, and this issue and the lack of external validation restrict the broader applicability of the model. Additionally, despite the application of the SMOTE algorithm, data imbalance may still lead to biased model performance in minority populations. To enhance the accuracy and stability of the model, research will aim to expand the sample size and involve multicenter validation in the future, ensuring the model’s generalizability. Finally, the follow-up duration in this study was limited to only 2 years, which may be insufficient to fully assess the long-term survival outcomes of patients with malignancies. Future studies will extend the follow-up period to evaluate the predictive performance and stability of the model over a longer time span.

## 5 Conclusions

In conclusion, our contrast-enhanced CT radiomics nomogram may be a dependable, precise, and noninvasive predictive tool to assess PFS in pediatric patients with PMMTs before surgery, with potential benefits for clinical decision-making and personalized treatment planning.

## Data Availability

The raw data supporting the conclusions of this article will be made available by the authors, without undue reservation.
